# Diagnostic profiles and predictors of treatment outcome among children and adolescents attending a national psychiatric hospital in Botswana

**DOI:** 10.1186/s13034-017-0144-9

**Published:** 2017-02-10

**Authors:** Anthony A. Olashore, Bechedza Frank-Hatitchki, Olorunfemi Ogunwobi

**Affiliations:** 1 Department of Psychiatry, University of Botswana Medical School, Gaborone, Botswana; 2 Sbrana Psychiatric Hospital, Lobatse, Botswana; 3 Department of Psychiatry, Bowen University Teaching Hospital, Ogbomoso, Nigeria

**Keywords:** Child and adolescent, Psychiatric disorders, Psychiatric hospital, Botswana

## Abstract

**Background:**

Attention is currently being drawn to child psychiatric care, most especially in the developed countries. This type of care is still rudimentary in the developing countries. Botswana is one of the African countries with good health care services but mental illness is given the low priority. Child and adolescent mental health care (CAMHC) is almost non-existent likely due to the dearth of research which would drive a policy change in this direction. Hence the need for this research as a step towards establishing a well-structured CAMHC.

**Objectives:**

To determine the pattern of presentation of child psychiatric disorders and the predictors of poor treatment outcome in the national psychiatric hospital in Botswana.

**Methods:**

This is a retrospective investigation comprising patients aged ≤17 years, consulting Sbrana Psychiatric Hospital over a 5-year period. It involves extraction of information from 238 patients’ records on socio-demographic characteristics, diagnosis and management.

**Results:**

The most common diagnosis was Attention deficit hyperactivity disorder (ADHD) with a prevalence of 25.2%. ADHD (60%) and Autism (58.3%) were more diagnosed in 5–9 years, whilst psychosis (80%) and depression (88.9%) amongst 14–17 years. Perinatal complication (OR 7.326, 95% CI: 1.312–40.899) and polypharmacy (OR 4.188, 95% CI: 1.174–14.939) independently predicted poor treatment outcome, after logistic regression.

**Conclusions:**

This study provided baseline information regarding children mental health in Botswana. It highlights the need for further research and to develop more specialized mental health care services for improved outcomes in children with mental health disorders.

## Background

In traditional African culture, it was previously assumed that mental illness “is unheard of” among children, (i.e., was inconceivable) [1], but recent epidemiological studies have revealed that psychiatric disorders are not only common but persistent, constituting about 30% of the global burden of illness in this age group [2–4]. Approximately, one in every five children and adolescents have a recognizable & treatable mental disorder and more than half of adult psychiatric disorders begin before age 15 [5–7]. Disorders most commonly encountered in both community and hospitals include epilepsy, conduct disorder (CD), anxiety/emotional disorders, mixed disorders of conduct and emotions, attention deficit hyperactivity disorders (ADHD), major affective disorders, pervasive developmental disorders, specific developmental disorders, psychoses, enuresis and mental retardation [8–10].

Pattern of presentation of child psychiatric disorders vary across different regions [8, 9]. In a study conducted in America, the most common diagnosis made was ADHD (43%), followed by CD (30%), while depressive disorders and Schizophrenia were 27 and 5% respectively [10]. Another study from Saudi Arabia revealed mental retardation with a prevalence (30.2%) and anxiety disorders (16%) as the most commonly encountered disorders [11], while Schizophrenia 50% and delirium (15%) were the most diagnosed in a Nigerian study [12]. Reasons for variation in presentation at different locations include age at presentation, delay in seeking help due to lack of awareness, poor socioeconomic status, waiting for more severe symptoms to appear, birth order and limited insurance coverage among others [8–12].

Pattern of presentation also varies according to age and gender, with diagnosis changing in individual patients with increasing age and frequently higher proportion of males than females [8–12]. Enuresis, feeding problems and developmental disorders are frequently seen in early childhood while Psychotic disorders such as schizophrenia rarely occur before age 14, but show a marked increase in prevalence after 15 years. Depression and drug abuse frequently start and are common in adolescence [5, 8–10, 13].

The effect of child disorders without early and adequate intervention are quite enormous and have serious consequences in their lives, the family and the society at large [9]. They commonly lead to underachievement, dependence or even delinquency depending on the type of disorder [8, 9]. Early recognition and prompt intervention have been shown to reduce mental health disease burden and improve quality of life in children and adolescents [14]. Nevertheless, studies from Europe and America have suggested some factors which to a large extent influence disease course and treatment outcome [15–17]. These include treatment adherence, family stability, polypharmacy, perinatal complication, nature of illness (externalizing versus internalizing) presence of co-morbid psychological/medical disorders; stressful life events, lack of specialized care and so on. These factors either influence treatment outcome directly or indirectly by influencing treatment adherence [11, 15–17].

Many of these factors are increasingly being addressed with the advent of specialized child and adolescent care, an improvement on the period when children with psychiatric disorders were being cared for by general adult psychiatrists [8, 9, 12]. Specialized child and adolescent care involves the use of a multidisciplinary care team which include child psychiatrists, child psychologists, speech therapists, social workers, neuropsychiatrists, educational occupational therapists among others and has greatly improved quality of care and reduced disease burden as well as treatment outcome [8, 9]. This type of care is still very rudimentary in the developing countries and the reasons for this are diverse [18]. Factors ranging from low socio-economic status, illiteracy and poor infrastructure are partly responsible [12, 19]. The impact of the perception in many African countries that childhood mental disorders are not medical conditions cannot be overemphasized [12]. Whilst some externalizing childhood mental disorders such as ADHD and CD are seen as “stubbornness,” with parents encouraged to resort to punitive corrective measures, Internalizing disorders such as autism and depression are linked to witchcraft with traditional or spiritual help being often sought. Botswana is not excluded from the usual African perception and practice of exhausting the traditional method of care before consulting the orthodox care, resulting in delayed presentation or presentation at the very severe state [20]. Of note is the “defective” family system which is characterized by non-marital childbearing, increasing number of female-headed households and the resultant poor family support. This has been shown to have negative effect on child health and plays a vital role in causing delay in help seeking [21].

Low priority for mental health care is another major factor which is not unconnected to the dearth of research to drive policies in favor of this field of medicine [22]. Botswana is among the middle income countries in Africa. It is rated 15th by the World Bank in terms of Gross National Income per capita (GNI). Its percentage of GDP on health care expenditure in 2013 was 5.4% which is lower than that of its neighbour South Africa, another middle income country with GNI rating of 12th and 8.8% total expenditure on health as percentage of GDP [23]. Services are available free for citizens at all levels of health care with 60.01% of funding for healthcare in Botswana being provided for by the government compared to the average for the African region of 48.5 [24]. In many other countries in Africa such as Nigeria, health care financing is mostly out of pocket [20, 24, 25]. However, mental illness is given the low priority in Botswana, with only 1% of the total health budget spent on mental health, compared to South Africa with up to 8% in some districts [23, 26]. This is further buttressed by World Health Organization report in 2011, which indicated that there were 0.25 general adult psychiatrists, 0.51 non-psychiatrists, 0.35 social workers and 1.52 psychologists per 100,000 population in Botswana [27].

Moreover, there is currently no child psychiatrist in Botswana, hence, quality mental health care for this group of individuals is almost non-existent. For the past five years, the only psychiatric facility in Botswana has been attending to the needs of children with mental disorders without any specialized care unit. This invariably implies that they are being seen together with adults; a type of care that is often associated with stigma, inadequate attention to health needs, and consequent poor treatment outcome [12]. Lack of data to prove the existence of child psychiatric disorders is largely responsible for this low priority given to child mental health and its attendant poor treatment outcome in the developing countries [18, 22]. We thus believe that, assessing the diagnostic profiles as well as factors influencing treatment outcome in the only mental health facility can not only inform a policy change in favor of CAMHC in Botswana, but also lay a foundation for a well-structured health care services for this group of people.

## Methods

### Study design and population

The study is a retrospective investigation, which involved extraction of information from the records of the patients (17 years and below) who attended Sbrana Psychiatric Hospital (SPH) between 1 January 2012 and 31 July 2016.

### Study location

SPH, Lobatse, is the only mental health referral hospital in Botswana and is government owned, which informed its use for thus study. It is a 300-bed facility located in the southern-eastern part of Botswana. The hospital offers both Out-patient and In-patient as well as day hospital care service. The hospital accepts all types of mental disorders, ranging from minor to the severe ones and serves as the only mental health referral facility for all the health institutions (private, public and all levels of health cares) in the country. The hospital provides for the psychiatric treatment of both adult and child mental and behavioral disorders. Other facilities available are psychology, sociology, occupational therapy, pharmacy, laboratory and community services.

### Sampling and data collection procedure

The hospital numbers of all the children and adolescents below 18 years were retrieved from the hospital computerized record system and used to retrieve patients’ files from the medical record library. A semi structured instrument was designed to assist in extraction of information from the case notes. These include information on the patient socio-demographic profile (age, gender, parents’ profiles, educational history and family history), clinical and management characteristics of the patients, such as, presenting symptoms, diagnostic classification patients’ management, and information on follow-up visits. SPH prides itself on proper documentation and a very good (computerized) record keeping which makes data extraction for research purposes easy. Moreover, clinical audits are conducted from time to time to ensure strict adherence to proper documentation. As a rule, all patients’ case files in SPH contain notes/input from every member of multidisciplinary team involved in patients’ care. These include, Birth records, reviews (psychiatric and medical), investigations, diagnoses, management and follow-up notes. Also included in all the files are case/discharge summaries with ICD-10 diagnoses.

All the researchers agreed on the designed pro-forma and all the information to be extracted from patients’ files, but only two of the researchers who are hospital specialists extracted the information. Every issue that needed clarification was discussed frequently and resolved. The two researchers who extracted the information worked together and agreed on the diagnosis, treatment outcome and any other sensitive information before they are finally entered into the instruments. Those records on which agreement could not be reached were excluded. This was done for all the records reviewed to avoid double coding.

The final and the major diagnoses were recorded. However, in those with co-occurring psychiatric disorders, the second and third diagnosis were recorded as multiple diagnoses. Treatment outcome was based on the agreement of the subjective remarks of the managing team which include the attending consultant psychiatrists, the psychologists, social workers, occupational therapists, psychiatric nurses and the relatives. These reports were majorly based on alleviation of symptoms and restoration of functions, as documented in the patients’ files. Outcome was one of these three possible options: Good (Improved) treatment outcome was used when most or all of the symptoms have subsided and patients’ functioning have either improved considerable or totally restored as assessed by the managing team. Poor treatment outcome was used when most of the symptoms were still present and the patient was unable to maintain adequate level of function particularly in the activities of daily living after at least 3 months of treatment. The third group comprise of those who either defaulted after the first visit or whose outcome could not be determined most especially due to poor documentation.

### Ethical considerations

Ethical approval was obtained from the University of Botswana ethical committee. Permission to access patients’ records was also sought from the ministry of health and the management of SPH.

### Data analysis

Data analysis was done using the Statistical Package for Social Sciences (SPSS for windows 16.0, SPSS Inc., Chicago, IL, USA). Frequency tables were employed for descriptive statistics such as socio-demographics, diagnosis and other clinical variables. Cross-tabulations were done to show the relationships between identified risk factors (socio-demographics and clinical variables) and treatment outcomes using Chi square test. The variables that were significantly associated with outcome were further entered into stepwise multiple regression analysis with backward elimination with poor treatment outcome as the dependent variable. The level of statistical significance was set at *p* < 0.05.

## Results

### Socio-demographics

The records of 238 of 251 patients aged 17 years and below, seen between 1 January 2012 and 31 July 2016 were extracted and analyzed. Thirteen case notes were totally left out due poor documentation. The mean age of the patients was 12.41(SD 4.1). Members of the male gender (60.5%) outnumbered that of their female (39.5%) counterparts. Majority (60.9%) of the patients came from the South and South-east district. Many of these children and adolescents came from families with 4 or less number of siblings (77.5%) and only 90 (39.6%) were the first-born. Of the 238 records extracted, only 200 had full documentation on parents’ marital status and these revealed that only 26 (13%) parents were married. In addition, only in 39 (17.6%) cases were both parents part of the patients’ care. The most common source of referral was from the parents/relatives accounting for 37.6%. Twenty-three (10.4%) were referred from the social welfare and the police while only sixteen (7.2%) were referred from the tertiary hospitals.

### Diagnosis

**Fig. 1:**
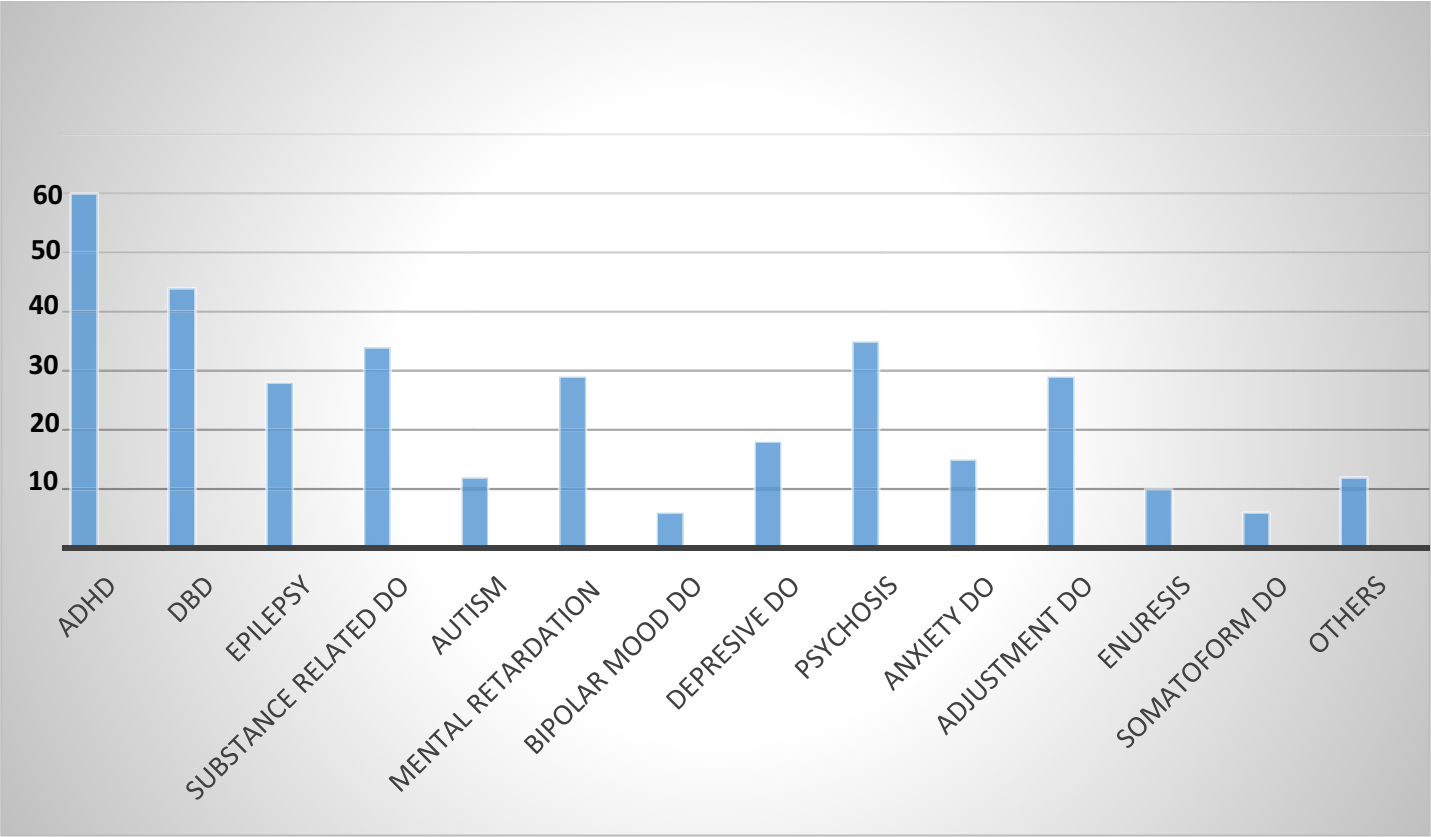
*DBD* disruptive behaviour disorder, *MR* mental retardation, *ADHD* attention deficit hyperactivity disorder, *DO* disorder, *Others* tic disorder, obsessive compulsive disorder, other stress related disorder such as acute stress reaction, posttraumatic stress disorders, organic disorders

Using ICD-10 diagnostic criteria, the final diagnosis of each patient was extracted from the hospital records. The most common overall diagnosis (including single and multiple) was ADHD 60 (25.2%) followed by disruptive behavior disorder (DBD) 44 (18.5%) (Fig. 1). One hundred and twenty-nine (54.2%) had single diagnosis, while the remaining had more than one diagnosis. ADHD was the commonest single diagnosis, accounting for 22.5% of the 129 with single diagnosis followed by adjustment disorder (14%), psychosis, including schizophrenia (11.6%) and DBD (8.5%). The three most commonly occurring pairs of diagnoses were ADHD and DBD (12%), substance related disorders and psychosis (9.2%) and ADHD and mental retardation (7.3%).

ADHD and Autism were significantly most frequently diagnosed in 5–9 years, whilst adjustment disorder, substance related disorders, psychosis, which includes schizophrenia and depression occurred most commonly amongst patients aged 14–17 years (Table 1). In the same vein, ADHD, autism and schizophrenia were commoner among males with depression and adjustment disorder common among females (Table 2). Only 18 (7.6%) had physical/medical co-morbidity of which the most common was speech and hearing impairment (33.3%), followed by congenital abnormality and physical deformity which were both 11.2%.

**Table 1:** Frequency of the overall diagnosis by age

Frequency of diagnosis	Age, N* (%)				Chi square	p value
	1–4	5–9	10–13	14–17		
	N = 10	N = 57	N = 50	N = 121		
ADHD	8 (13.3)	36 (60.0)	11 (18.3)	5 (8.3)	88.241	<*0.01*
DBD	3 (6.8)	15 (34.1)	11 (25.0)	15 (34.1)	7.041	0.061*
Epilepsy	1 (3.6)	4 (14.3)	4 (14.3)	19 (67.9)	3.412	0.312*
Substance related *do*	0 (0)	0 (0)	1 (2.9)	33 (97.1)	36.603	<0.01*
Autism	1 (8.3)	7 (58.3)	2 (16.7)	2 (16.7)	9.426	0.014*
Mental retardation	1 (3.4)	10 (34.5)	9 (31.0)	9 (31.0)	5.954	0.096*
Bipolar mood *do*	0 (0)	0 (0)	2 (33.3)	4 (66.7)	2.274	0.506*
Depressive *do*	0 (0)	1 (5.6)	1 (5.6)	16 (88.9)	9.822	0.015*
Psychosis	0 (0)	1 (2.9)	6 (17.1)	28 (80.0)	17.409	0.001*
Anxiety disorder	0 (0)	3 (18.8)	6 (37.5)	7 (43.8)	2.483	0.447*
Adjustment *do*	0 (0)	2 (6.9)	5 (17.2)	22 (75.9)	9.042	0.023*
Enuresis	0 (0)	4 (40.0)	4 (40.0)	2 (20.0)	5.148	0.135*
Somatoform *do*	0 (0)	0 (0)	4 (66.7)	2 (33.3)	5.930	0.066*
Others	1 (8.3)	5 (41.7)	2 (16.7)	4 (33.3)	3.587	0.279*

*do* disorder, *DBD* disruptive behavior disorders include conduct disorder and oppositional defiant disorder, *ADHD* attention deficit hyperactivity, *psychosis* schizophrenia and other psychotic *do*, Others Tic *do*, obsessive compulsive *do*, stress related *do*, organic *do*

* Fisher’s exact test

N^*^ = 238. Significant relationships in italics

**Table 2:** Frequency of the overall diagnosis by gender

Frequency of overall diagnosis	Gender		Chi square	p value
	Male N (%)	Female N (%)		
ADHD	47 (78.3)	13 (21.7)	10.672	*0.01*
DBD	31 (70.5)	13 (29.5)	2.236	0.135
Epilepsy	15 (53.6)	13 (46.4)	0.638	0.424
Substance related *do*	28 (82.4)	6 (17.6)	7.924	*0.005*
Autism	11 (91.7)	1 (8.3)	5.135	0.031*
Mental retardation	24 (82.8)	5 (17.2)	6.844	*0.009*
Bipolar mood *do*	3 (50.0)	3 (50.0)	0.284	0.683*
Depressive *do*	3 (16.7)	15 (83.3)	15.660	<0.01*
Psychosis	27 (77.1)	8 (22.9)	4.754	*0.029*
Anxiety disorder	5 (31.2)	11 (68.8)	6.143	*0.013*
Adjustment *do*	9 (31.0)	20 (69.0)	12.002	*0.001*
Enuresis	7 (70.0)	2 (30.0)	0.408	0.744*
Somatoform *do*	2 (33.3)	4 (66.7)	1.902	0.174*
Others	7 (58.3)	5 (41.7)	0.25	0.875

*do* disorder, *DBD* disruptive behavior disorders include conduct disorder and oppositional defiant disorder, *ADHD* attention deficit hyperactivity, *psychosis* schizophrenia and other psychotic *do*, Others Tic *do*, obsessive compulsive *do*, stress related *do*, organic *do*

* Fisher’s exact test

N^*^ = 238. Significant relationships in italics

### Identified risk factors and treatment outcome

Of the 238 patients, 109 (45.8%) had improved as at the last time they were reviewed, 78 (32.8%) had poor treatment outcome, while the remaining 51 (21.4) were excluded from further analysis either due to incomplete records or default after first visit. Seventy-seven (70.6%) of those who had good treatment outcome were 11 years and above (χ^2^ = 6.382, *p* = 0.012). Male patients were more likely to have poor treatment outcome compared to their female counterparts (χ^2^ = 4.343, *p* = 0.037). Those who had perinatal complications or early childhood illness were more likely to have poor outcome than those with uneventful perinatal history (χ^2^ = 4.937, *p* = 0.026) or without early childhood illness (χ^2^ = 4.218, *p* = 0.040). Other factors that were associated with poor treatment outcome include out-patient mode of care (χ^2^ = 31.072, *p* < 0.01) and poly-therapy (χ^2^ = 7.197, *p* = 0.007). Specialist (general psychiatrist) care on the other hand was associated with good treatment outcome (χ^2^ = 7.238, *p *= 0.007) (Table 3).

**Table 3:** The relationship between identified risk factors and treatment outcome

Risk factors	Outcome N^*^ (%)		*df*	*χ* ^*2*^	*p*
	Good	Poor			
Age group					
≤10	32 (46.4)	37 (53.6)	1	6.382	*0.012*
≥11	77 (65.3)	41 (34.7)			
Gender					
Female	47 (68.1)	22 (31.9)	1	4.343	*0.037*
Male	62 (52.5)	56 (47.5)			
No of sibling					
4 or less	86 (59.3)	59 (40.7)	1	0.133	0.715
5 or more	19 (55.9)	15 (44.1)			
Order of birth					
First born	37 (50.7)	36 (49.3)	1	3.233	0.072
Others	68 (64.2)	38 (35.2)			
Family type					
Same parents	38 (63.3)	22 (36.7)	1	1.371	0.242
Different parents	60 (54.1)	51 (45.9)			
Care giver					
Both parents	18 (51.4)	17 (48.6)	1	0.622	0.430
Single parent and others	80 (58.8)	56 (41.2)			
Past psychiatric history					
Absent	23 (71.9)	9 (28.1)	1	2.931	0.087
Present	86 (55.5)	69 (44.5)			
Medical history					
Absent	102 (58.4)	73 (41.7)	1	0.000	0.997
Present	7 (58.3)	5 (41.7)			
Family history					
Absent	67 (58.8)	47 (41.2)	1	0.016	0.901
Present	37 (57.8)	27 (42.2)			
Perinatal complication					
Absent	87 (61.7)	54 (38.3)	1	4.937	*0.026*
Present	9 (37.5)	15 (62.5)			
Early childhood illness					
Absence	71 (64.0)	40 (36.0)	1	4.218	*0.040*
Presence	26 (47.3)	29 (52.7)			
Psychiatric co-morbidity					
Absent	60 (61.9)	37 (38.1)	1	1.055	0.304
Present	49 (54.4)	41 (45.6)			
Physical co-morbidity					
Absent	99 (57.2)	74 (42.8)	1	1.075	0.300
Present	10 (71.4)	4 (28.6)			
Mode of care					
In-patient	43 (93.5)	3 (6.3)	1	31.072	<*0.01*
Out-patient	66 (46.8)	75 (53.2)			
Type of intervention					
Only Pharmacological	19 (51.4)	18 (48.6)	1	0.913	0.339
Only Psychological or both	90 (60.0)	60 (40.0)			
Specialist (General psychiatrist) care					
Given	90 (63.8)	51 (36.2)	1	7.238	*0.007*
Not given	19 (41.3)	27 (58.7)			
Prescribing pattern					
Monotherapy	50 (71.4)	20 (28.6)	1	7.197	*0.007*
Poly-therapy	14 (43.8)	18 (56.2)			

*χ* ^*2*^ Chi square, *df*   degree of freedom, *p* p value

N^*^ = N not equal to 238 due to missing data; only those with good (improved) and Poor treatment outcome (187) were analysed, those who defaulted after the first visit (51) were excluded from analysis. Significant *p* value in italics

### Multiple regression analysis of the risk factors for poor treatment outcomes

The identified risk factors that were significant on bivariate analysis were entered into a multiple regression model with backward elimination which involved 4 steps. Early childhood illness and gender were the first variables to exit the model at step 2 and 3 respectively. Age group, place of management and specialist were also eliminated from step 4 of the regression model. The remaining two independent variables: namely perinatal complication (OR 7.326, *p* = 0.023, 95% CI: 1.312–40.899) and prescribing pattern (OR 4.188, *p* = 0.027, 95% CI: 1.174–14.939) were produced by the model (Table 4). The model summary revealed a Nagelkerke R^2^ of 0.519 and thus implies that these two variables explains 51.9% of the variance in predicting poor treatment outcome.

**Table 4:** Multiple regression analysis of the risk factors for poor treatment outcomes

Risk factors	P value	OR	95% CI	
			Lower	Upper
Step1				
Age group^a^	0.349	1.830	0.516	6.490
Gender^b^	0.353	1.888	0.493	7.223
Perinatal complication^c^	0.027	7.861	1.271	48.620
Early childhood illness^d^	0.759	0.815	0.221	3.013
Place of management^e^	0.998	2.854E9	0.000	–
Specialist care^f^	0.147	3.679	0.633	21.385
Prescribing pattern^g^	0.016	5.235	1.361	20.140
Step 2				
Age group	0.294	1.925	0.566	6.552
Gender	0.360	1.868	0.490	7.118
Perinatal complication	0.028	7.428	1.246	44.278
Place of management	0.998	2.772E9	0.000	–
Specialist care	0.114	3.954	0.718	21.779
Prescribing pattern	0.017	5.136	1.346	19.597
Step 3				
Age group	0.379	1.711	0.517	5.661
Perinatal complication	0.019	8.335	1.414	49.144
Place of management	0.998	3.525E9	0.000	–
Specialist care	0.122	3.688	0.706	19.265
Prescribing pattern	0.021	4.780	1.265	18.062
Step 4				
Specialist care	0.088	4.110	0.809	20.881
Perinatal complication	*0.023*	*7.326*	*1.312*	*40.899*
Prescribing pattern	*0.027*	*4.188*	*1.174*	*14.939*

*OR*  odd ratio, *CI*  confidence interval

Significant test of association in italics. Nagelkerke R^2^ = 0.519

^a^ ≥11 years

^b^Female

^c^Absent

^d^Absent

^e^In-patient care

^f^Given

^g^Monotherapy

## Discussion

The results of the study demonstrated that childhood psychiatric disorders are clearly present in Botswana as in the rest of Africa [12, 18, 29] and they are being recognized and referred for psychiatric care. It is noteworthy that between January 2012 and July 2016, only 251 patients in the child and adolescent age range were recorded. This implies that the belief that childhood psychiatric disorder “is unheard of” still exists in Botswana. Although there is no published data to substantiate the possibility of underutilization of child psychiatric services when compared to the general adult service in the same facility, the hospital records revealed that an average of 100 new adult cases presented in a year. Moreover, in a population of over 2million with approximately 43% below the age of 19 years [28], this figure demonstrates the probability of significant unmet need of child and adolescent mental health. Nonetheless, a community study would be required to establish the level of awareness and service utilization.

The mean age of 12.41 years is consistent with those of the American study and a study from West Africa [10, 29]. The age range was 2–17 years and the upper limit of 17 year is in agreement with a West African study, where patient who are already 18 years are being treated as adult [29]. Male preponderance was noted in this group, as in many other studies in children, including the community based ones [2, 3, 8, 9]. The overrepresentation (60.9%) of those from the South-west and Southern part of Botswana may simply be a reflection of the location of the facility and thus suggests a skewness in the coverage of the facility and its community outreach programs.

It is not surprising that most of the informants/caregivers were mother comprising of 57.1% of the 182 single parent or others, because of the increasing emergence of non-marital child bearing and female headship in Botswana [21]. In the same vein, a large proportion (37.6%) of patients presented without any formal referral, a probable effect of the community mental health outreach of the hospital. Despite its drawbacks which is majorly due to shortage of personnel and the fact that it was mostly targeted at the general adult mental health [20], it may have positively influenced the awareness of child mental health among parents and families who have benefited from the hospital services through snow-balling.

Externalizing disorders such as ADHD and DBD presented relatively more often, followed by Psychotic disorders. While our study agrees to an extent with that of the American study where ADHD and DBD were predominant [10], it differs from a study in Nigeria whose commonly encountered disorder was schizophrenia [12]. This may be a result of the comprehensive free health program available for all citizens of Botswana as against out of pocket payment which predominates in Nigeria and many other African countries [25]. The absence of a financial barrier to seeking care may help presentation at the clinical setting to more closely mirror the prevalence in the community. In Botswana, over 60% of total healthcare funding is provided by the government with only about 5% funding being out of pocket payment as compared to about 23% of government funding and almost 73% out of pocket funding in Nigeria [23]. In addition, the mean age in the current study and the American were 12.41 and 11.9 respectively, unlike in the Nigerian study (16.38) where schizophrenia and other disorders more specific to the older age group are expected. The availability of a government funded free health program removes a critical barrier to help seeking. This in turn enables parents to seek professional help for childhood behavioral disorders like ADHD and DBD, which might have otherwise been construed as “stubbornness”. Moreover, it is not unexpected that childhood disorders of externalizing type are by nature disruptive and easier to identify as problems. Nonetheless, it will be necessary to compare this result with a community based study in the same population to determine if our finding is a true reflection of the incidence or due to lack of identification of other disorders.

The age related frequency of diagnoses demonstrate that the presentation follows a similar pattern to the known age of onset of childhood psychiatric disorders [8–10] with ADHD presenting more commonly in age 5–9 years (χ^2^ = 88.241; *p* < 001), while other known disorders more commonly seen in adolescents as compare to early childhood such as depressive disorders (FET = 9.822; *p* = 0.015), substance related disorders (FET = 36.603; *p* < 0,01) and psychosis such as schizophrenia (FET = 17.409; *p* = 0.001) presented more often within 14–17 years [8–10, 12]. A pattern of gender distribution similar to what has been documented was also seen, with ADHD, autism and schizophrenia being more often diagnosed among males, while depressive disorders and anxiety disorders were diagnosed more often among females [8–10, 22]. This pattern may indirectly indicate that the relative presentations mirror the pattern in the community. It is however impossible to come to this conclusion until a community study is done to compare the findings.

We found that those who were above 10 years were more likely to achieve a good treatment outcome compare to those who were 10 and below (χ^2^ = 6.382, *p* = 0.012). Possibly, the higher success rate achieved in the older age group may be due to the fact that the disorder most commonly presented at these age group were the same as those found in adults, for which the available specialist were specifically trained for, since they are all general adult psychiatrist. In a similar manner, gender is seen to be significantly associated with the outcome, with female gender having higher rates of good treatment outcome. This may be explained by the male preponderance of chronic childhood and adolescent behavioral disorders including ADHD (78.3%), Autism (91.7%), mental retardation (82.8%) and substance related disorders (82.4%) as compared with the female preponderance of emotional disorders such as anxiety disorders (68.8%) and adjustment disorders (69.0%). In addition males had a higher proportion of diagnosis of psychoses (77.1%). This distribution may simply be as a result of identification bias. It has previously been reported that the female gender present less disruptive symptoms than their male counterpart even within diagnostic categories [30]. It is noteworthy however that gender disparity in the presentation and diagnoses of mental disorders have been noted in adult populations [31–33]. Nevertheless, neither age nor gender significantly contributed to the prediction of poor treatment outcome after a multiple regression analysis as reported by a more recent study with similar design [11, 29].

One would expect a significant relationship between family characteristics such as family type, number of siblings, parent’s marital status, and poor treatment outcome as documented in previous reports [8, 9, 34]. Similar to what was reported by Al-Habeeb et al., we did not observe any association between these variables, possibly because heath care provision in Botswana is free for all the citizens at all levels including tertiary level of care. This may have significantly reduced the burden of care on the family.

The mode of care, whether in-patient or out patient is largely dependent on the age, severity and the type of disorder [8]. The older patient with severe disorders such as schizophrenia and depression are more likely to be admitted while younger children with ADHD and autism were more likely to have out-patient mode of care [8, 9, 12]. In the current study, 44 out of 46 who had inpatient care were above 10 years and thus fell into the category of those with disorder similar to the general adult psychiatry which the hospital is adequately equipped for. This may partly explain the association observed between in-patient care and good outcome (χ^2^ = 31.07, *p* < 0.01) in this study. In addition, patient on admission can easily be monitored and may not be discharged until they have improved. Notwithstanding this association with bivariate analysis, mode of care does not explain any variance in the regression model similar to the report of previous authors [8, 9, 11].

Studies have established an association between perinatal complication early childhood illnesses and various child and adolescent psychiatric disorders. Some of these complications cause permanent damage to the brain which may present with psychiatric disorders especially when they later encounter adverse psychosocial events [8, 9]. These complication at times may be very elusive or difficult to detect, thus making some psychiatric disorder very difficult to treat. It is therefore not astounding that we found an association between perinatal complication and poor treatment outcome (χ^2^ = 4.937, *p* = 0.026) as in previous reports [8, 9, 11]. Those who reported perinatal complication were 7 times more likely to have a poor treatment outcome in this study (OR 7.326, 95% CI: 1.312–40.899). This suggests the need for more specialized care which involves looking beyond psychiatric manifestation of possibly undetected organic damage. Even though one may not expect the same level of improvement as in those without brain damage, but a more specialized care would improve functioning as well as quality of life [8, 9].

Another variable that contributed significantly to the prediction of poor treatment outcome is the prescribing pattern. The current study revealed that, those that were treated with more than one medication at a time were 4 more times likely to have a poor treatment outcome (OR 4.188, 95% CI: 1.174–14.939). Psychiatric polypharmacy has been defined as the prescription of two or more psychiatric medications concurrently to a patient [35, 36]. This has been described in the elderly [37] and in children [38]. More than a quarter (31.4%) of the patient on pharmacotherapy in the current study are in this category and a significant number of them had a poor treatment outcome (χ^2^ = 7.197, *p* = 0.007). The reasons for polypharmacy have been widely discussed [35, 36]. The practice could be that a therapist finds that the administration of a single medication was ineffective in treating psychiatric symptoms [35, 36]. It could be to treat side/adverse effects, co-morbid psychological or physical symptoms, and diagnostic dilemma, among others. Although one could not pin-point the reasons for polypharmacy amongst our sample, but the presence of co-morbid psychological disorder (45%) which may be related to perinatal complication is suggestive.

Polypharmacy has been shown to be associated with poor treatment outcomes for the following reasons: increased vulnerability to adverse reactions, poor compliance and drug interaction, induction of liver enzyme which may reduce bioavailability of the major drugs [39, 40]. Clearly, there are some times when polypharmacy is necessary, particularly in the treatment of adverse/side effect of the major medication and co-morbidity. Its negative effect on treatment outcome can be significantly addressed through rational prescribing or using the concept of “personalized medicine” [35]. This further highlights the need for specialized child and adolescent mental health care in the country, where children will only be attended to by those who are specially trained to identify their need and deliver a tailored mental health care to them.

## Recommendations

Following the findings of this study, a specialized training is recommended for interested members of professional staff whose services will be dedicated only to children and adolescents’ mental health care and research. The current drive towards increasing awareness of mental health disorders and treatment should be sustained and strengthened. Particular attention should be given to increasing the geographical spread of the awareness programs and increasing its focus on child and adolescent mental health disorders. Adequate management of perinatal period is advocated as a preventive measure for mental disorders in children and adolescents.

## Limitations and strengths

This study highlights the pattern of psychiatric disorder and the factors that influence the outcome of service delivery to the children and adolescent in the only psychiatric referral hospital in Botswana. Thus the findings must be interpreted with caution, owing to the reliance on the hospital record and the reports of the managing team which could be subjective. The generalizability of the study to the general population is also limited being a hospital-based study. Nevertheless, consistent rules were used in the selection of the samples and this screened out incomplete records which were either controversial or not informative. All the authors communicated from time to time and agreed on these rules, but the extraction was done by two of the hospital consultants (psychiatric specialists) who were part of the study. In addition, only the results that were agreed upon by all the members of the managing team were used for the analysis.

## Future research

Our study suggests a possibility of low psychiatric service utilization in Botswana, however, this is difficult to establish without a community study to compare with, thus indicating a need for community studies.

The Nagelkerke R^2^ = 0.519 indicates a moderately strong relationship between the predictors and the prediction. In other words, the two independent variables namely: perinatal complication and polypharmacy explain 51.9% of the variance in predicting a poor treatment outcome. This perhaps suggests that other factors which comprise of the remaining 48.1% related to the poor treatment outcome are yet to be investigated. These factors may include type and nature of psychiatric disorders, adherence, and other socio-cultural factors which may form the subject for further research. Other relevant research questions may include the sustainability of treatment under the comprehensive free medical coverage and quality and types of medications being used.

## Conclusions

This study has provided baseline information regarding child and adolescent mental health in Botswana. It provided a broad idea of the commonly encountered child psychiatric disorder by age and sex in the only mental referral hospital in Botswana.

Only two (perinatal complication and polypharmacy) of all the risk factors associated with poor treatment outcome emerged as its independent predictors. Whilst the non-modifiable factor namely perinatal complication suggests the need to improve our antenatal care, polypharmacy indicates the need for more specialized care for children with mental disorders.

Finally, our study highlights the need for further research in this psychiatric subspecialty for improved outcomes in children and adolescents with mental health disorders.

## Abbreviations

ADHD: attention deficit hyperactivity disorder
CD: conduct disorder
DBD: disruptive behaviour disorder
CAMHC: child and adolescent mental health care
SPH: Sbrana Psychiatric Hospital
GNI: gross national income
GDP: gross domestic product
